# Trends in anemia care in non-dialysis-dependent chronic kidney disease (CKD) patients in the United States (2006–2015)

**DOI:** 10.1186/s12882-018-1119-7

**Published:** 2018-11-09

**Authors:** Haesuk Park, Xinyue Liu, Linda Henry, Jeffrey Harman, Edward A. Ross

**Affiliations:** 1 Department of Pharmaceutical Outcomes and Policy, University of Florida College of Pharmacy, HPNP Building Room 3325, 1225 Center Drive, Gainesville, FL 32610 USA; 2 Department of Behavioral Sciences and Social Medicine, Florida State University, College of Medicine, Tallahassee, FL 32306 USA; 3 Department of Internal Medicine, University of Central Florida, College of Medicine, Orlando, FL 32827 USA

**Keywords:** Erythropoiesis-stimulating agent (ESA), Chronic kidney disease (CKD), Anemia, FDA safety warnings

## Abstract

**Background:**

The objective of the study was to examine overall anemia management trends in non-dialysis patients with chronic kidney disease (CKD) from 2006 to 2015, and to evaluate the impact of Trial to Reduced Cardiovascular Events with Ananesp Therapy (TREAT)‘s study results (October 2009) and the US Food and Drug Administration (FDA)’s (June 2011) safety warnings and guidelines on the use of ESA therapy in the current treatment of anemia.

**Methods:**

A retrospective cohort analysis of anemia management in CKD patients using Truven MarketScan Commercial and Medicare Supplemental databases was conducted. Monthly rates and types of anemia treatment for post-TREAT and post-FDA safety warning periods were compared to pre-TREAT period. Anemia management included ESA, intravenous iron, and blood transfusion. A time-series analysis using Autoregressive Integrated Moving Average (ARIMA) model and a Generalized Estimating Equation (GEE) model were used.

**Results:**

Between 2006 and 2015, CKD patients were increasingly less likely to be treated with ESAs, more likely to receive intravenous iron supplementation, and blood transfusions. The adjusted probabilities of prescribing ESAs were 31% (odds ratio (OR) = 0.69, 95% confidence interval (CI): 0.67–0.71) and 59% (OR = 0.41, 95% CI: 0.40, 0.42) lower in the post-TREAT and post-FDA warning periods compared to pre-TREAT period. The probability of prescribing intravenous iron was increased in the post-FDA warning period (OR = 1.11, 95% CI: 1.03–1.19) although the increase was not statistically significant in the post-TREAT period (OR = 1.03, 95% CI: 0.94–1.12). The probabilities of prescribing blood transfusion during the post-TREAT and post-FDA warning periods increased by 14% (OR = 1.14, 95% CI: 1.06–1.23) and 31% (OR = 1.31, 95% CI: 1.22–1.39), respectively. Similar trends of prescribing ESAs and iron supplementations were observed in commercially insured CKD patients but the use of blood transfusions did not increase.

**Conclusions:**

After the 2011 FDA safety warnings, the use of ESA continued to decrease while the use of iron supplementation continued to increase. The use of blood transfusions increased significantly in Medicare patients while it remained stable in commercially insured patients. Results suggest the TREAT publication had effected treatment of anemia prior to the FDA warning but the FDA warning solidified TREAT’s recommendations for anemia treatment for non- dialysis dependent CKD patients.

**Electronic supplementary material:**

The online version of this article (10.1186/s12882-018-1119-7) contains supplementary material, which is available to authorized users.

## Introduction

The Centers for Disease Prevention and Control (CDC) estimate that one in every ten adults in the US are currently living with chronic kidney disease (CKD) with varying levels of severity [1]. Patients with moderate to severe cases of CKD typically develop anemia (National Kidney Foundation definition of anemia: adult males < 13.5 g/dL and adult females < 12.0 g/dL) treated with iron supplements, erythropoiesis stimulating agents (ESAs), and or blood transfusions dependent on patient symptoms [2, 3].

In 1997, the first set of comprehensive guidelines for the treatment of CKD-associated anemia was published by the National Kidney Disease Outcomes Quality Initiative (KDOQI) [4]. In these guidelines, KDOQI endorsed using ESAs to avoid exposure to blood transfusions especially in potential transplant candidates [4]. However, in recent years, several studies raised concerns about the use of ESA’s in treating anemia among CKD non-dialysis patients.

Three studies in particular, CHOIR (Correction of Hemoglobin and Outcomes in Renal Insufficiency, 2006) [5], CREATE (Cardiovascular Risk Reduction by Early Anemia Treatment with Epoetin Beta, 2006) [6], and TREAT (Trial to Reduced Cardiovascular Events with Ananesp Therapy, 2009) [7], demonstrated that ESA’s failed to reach their stated endpoints of reducing mortality and cardiovascular events in CKD patients [5–7]. In addition, the landmark study, TREAT, reported that treatment with darbepoetin did not reduce mortality or cardiovascular events, but its use resulted in a 2-fold higher stroke rate in CKD patients not undergoing dialysis [7].

Though there were significant design differences between the three studies, the results of all studies suggested that aiming for higher hemoglobin levels with higher doses of ESA agents may be the main contributors to the adverse outcomes of these studies. On the negative side, patients who received placebo agents were exposed significantly more to red blood cell transfusions and its associated risks [8].

Nonetheless, as a result of these studies, regulatory and reimbursement changes occurred which reduced the suggested ESA dose and lowered the target hemoglobin concentrations. Following these study results in June 2011, the US Food and Drug Administration’s (FDA) officially changed ESA’s labeling to recommended that the ESA should no longer be prescribed to obtain a target hemoglobin concentration range (10 to 12 g/dL), but should only be used for patients whose hemoglobin concentrations were below 10 g/dL, were symptomatic, and to avoid blood transfusions [9], which was followed by Kidney Disease Improving Global Outcomes (KDIGO) clinical practice guideline in 2012 [10].

However, little is known about the trends of anemia treatment in patients with non-dialysis-dependent CKD following the release of TREAT study results and the 2011 FDA guidelines. Therefore, this study sought to (1) examine overall anemia management trends (ESA, intravenous iron, and blood transfusion) in non-dialysis patients with CKD from 2006 to 2015, and (2) to specifically evaluate the impact of TREAT’s study results (October 2009) and FDA’s (June 2011) safety warnings and guidelines on the use of ESA therapy in the current treatment of anemia.

## Methods

### Data source

We conducted a retrospective cohort study using the Truven Health Analytic Marketscan Commercial database and the Medicare Supplemental database (January 2005 through September 2015). The Commercial Database contains records on employer-sponsored insurance covering 15–50 million individuals annually. The Medicare Supplemental database contains records on retired employees and their spouses who are enrolled in Medicare with supplemental insurance paid for by their former employers, representing ~ 2.5 million covered individuals annually. The Medicare Supplemental Database include both the Medicare covered and employer-paid portions of healthcare encounters utilization, and cost information. Institutional review board approval was obtained from the University of Florida.

### Study population

To conduct a trend analysis, we established monthly cohorts of patients who met the inclusion criteria from January 2006–September 2015 (116 months of study =116 cohorts). 2005 data was used to ensure at least 12-months of baseline period. Patients entered the cohort if they: 1) were at least 18 years of age; 2) had at least two outpatient claims or one inpatient claim for CKD, defined as International Classification of Diseases, Ninth Revision, 585.3 (stage 3), 585.4 (stage 4), 585.5 (stage 5) within 1-year period, or at least one specific (585.3–585.5) and one unspecific claim (585.9) for CKD; and 3) were continuously enrolled for at least 1-year before they entered the cohort. Among CKD patients, if a least one claim carried a CKD stage code during the 1-year baseline period that defined CKD, the code for the highest stage was used each month. Patients were excluded if they had a diagnosis code(s) for any malignancy as ESAs are also used to treat anemic cancer patients undergoing chemotherapy. Patients were censored when they began dialysis, received a kidney transplant, end of enrollment, or 30 September, 2015, whichever came first. For each monthly cohort, denominator was calculated as the number of patients who were alive and were not censored the entire month; and numerator was calculated as the number of patients who received anemia care (defined below).

To examine the association between the publication of TREAT (October 2009), FDA safety warning on ESAs (June 2011) and anemia therapy in CKD patients, the study period was divided into three periods. Specifically, the first period, the pre-TREAT period, covers the 45 months from January 2006 to September 2009 before the TREAT study was published. The second period, the post-TREAT period, encompassed the 20 months after the TREAT study was published and ended right before the June 2011 new FDA guidelines were released (from October 2009 through May 2011). The third period, the post-FDA warning period, included the 51 months from the publication of the FDA ESA guidelines to the end of the study (from June 2011 through September 2015).

### The use of ESA, intravenous iron, and blood transfusions

ESA therapy was defined as the receipt of darbepoietin alfa and/or epoetin alfa. ESA therapy was identified using Healthcare Common Procedure Coding System (HCPCS) for the inpatient and outpatient settings, and National Drug Code (NDC) for the outpatient pharmacy claims. Receipt of intravenous iron was identified using HCPCS codes. Blood transfusions was identified using Clinical Procedural Terminology (CPT), HCPCS codes, or ICD-9 procedure codes. (see Additional file 1: Table S1 for a complete list of codes). We calculated the monthly anemia treatment rates as the proportion of patients with at least one prescription claim of ESA, intravenous iron or blood transfusions divided by the total number of CKD patients each month.

### Covariates

Baseline patient characteristics were obtained from the 12-month period prior to the entrance of cohort each month, including demographic characteristics (i.e., age, gender, region), the Charlson comorbidity Index (CCI), presence of chronic diseases (i.e., diabetes mellitus, hypertension, heart failure, cardiovascular disease, peripheral artery disease, chronic obstructive pulmonary disease (COPD), and the involvement of a nephrologist (> = 2 visits in the previous 12 months). The presence of chronic diseases was defined as the presence of one inpatient or two outpatient claims within 12-month period before the cohort.

### Statistical analysis

Patient characteristics were grouped into one of the three time periods (pre-TREAT, post-TREAT, post-FDA warning period) previously described. We then tabulated and plotted receipt of each category of anemia treatment by insurance type (Medicare and commercial insurance) and by CKD stages for ESA use only. To describe the general trend in anemia management from January 2006 – September 2015, we used Autoregressive Integrated Moving Average (ARIMA) model. ARIMA is a well-established modeling strategy for time series data with repeated observations. Since the series were not stationary, first-order differences in proportion of patients receiving treatment were used for modeling to achieve stationarity. The autocorrelation and partial autocorrelation functions of the complete differenced series were used to identify the ARIMA models. We included autoregressive (AR) terms, moving average (MA) terms, and seasonality in the model and used Akaike Infomration Criteria (AIC) to select the most parsimonious model.

To explore the association of the publication of TREAT and/or FDA safety warnings with anemia management in CKD, we used a Generalized Estimating Equation (GEE) model with a binominal distribution and logit-link function. The GEE model with autoregressive correlation structure was employed to account for patient-level factors for ESA prescribing, intravenous iron prescribing, and blood transfusions as well as repeated measurement. The GEE model estimated the probability of receiving anemia treatment on a monthly basis during the post-TREAT and post-FDA safety waning periods compared to the pre-TREAT period. All statistical analyses were 2-tailed, with an a priori significance level of α = 0.05. All analyses were conducted using SAS 9.4 (SAS Institute Inc., Cary, NC) and STATA version 14 (Stata Corp., College Station, TX).

## Results

### Patient characteristics

We identified 157,293 Medicare and 361,385 commercially insured unique patients between 2006 January and September 2015 who had CKD and were not on dialysis. Table 1 summarizes the baseline demographic and clinical characteristics of the study population during the three study periods using the median month of each study period. Medicare patients were older, more likely to be women, and more likely to have CKD stages 4 and 5, and a higher CCI score relative to commercially insured patients. Patient demographics including age and gender were comparable in the three periods. Compared to the pre-TREAT period, patients in the post-TREAT and post-FDA warning periods tended to have more comorbidities including hypertension, heart failure, peripheral artery disease, and COPD but less advanced CKD diseases.

**Table 1:** Demographic and clinical characteristics of the study populations

	Medicare			Commercial Insurance		
	Pre-TREAT ^a^	Post-TREAT ^b^	Post- FDA warning ^c^	Pre-TREAT ^a^	Post-TREAT ^b^	Post- FDA warning ^c^
No. unique patients	48,614	64,694	117,452	103,980	127,054	244,191
No. patients in the median month of each period	12,806	29,594	39,310	22,559	50,433	55,218
Male (%)	52.1	49.8	49.4	55.2	55.6	56.3
Age 19–44 (%)	0	0	0	13.3	12.1	11.4
Age 45–64 (%)	1.2	1.0	1.2	85.6	85.1	84.9
Age > =65 (%)	98.8	99.0	98.8	1.1	2.8	3.6
CKD stage 3	43.4	49.0	51.0	70.0	76.5	80.8
CKD stage 4	49.6	45.8	44.2	24.3	19.5	15.9
CKD stage 5	7.0	5.2	4.9	5.7	4	3.4
Diabetes mellitus (%)	43.8	47.7	51.2	43.9	42.6	41.8
Hypertension (%)	52.7	73.2	85.9	62.9	73.1	73.1
Heart Failure (%)	10.1	17.4	21.2	5.2	6.2	6.4
Cerebrovascular disease (%)	14.7	16.9	18.4	5.5	5.5	5.0
Peripheral Artery Disease (%)	8.8	13.5	16.2	4.2	4.9	4.3
Chronic obstructive pulmonary disease (%)	16.3	19.7	23.1	6.8	8.3	8.1
Nephrologist involvement (%)	46.8	45.3	39.0	49.0	48.0	44.8
Charlson Comorbidity Index, mean (SD)	4.2 (1.8)	4.6 (2.0)	4.9 (2.1)	3.7 (1.8)	3.7 (1.8)	3.7 (1.8)

^a^ January 2006 through October 2009; November 2007 cohort was used to summarize demographics and clinical characteristics of CKD patients during the pre-TREAT periods

^b^ November 2009 through June 2011; August 2010 cohort was used to summarize demographics and clinical characteristics of CKD patients during the post-TREAT periods

^c^ July 2011 through September 2015; August 13 cohort was used to summarize demographics and clinical characteristics of CKD patients during the post-FDA warning periods

### Autoregressive integrated moving average (ARIMA) model

Additional file 1 : Table S2 shows ARIMA model results for ESA, intravenous iron supplementation, and blood transfusion. (See : Additional file 1: Tables S3 and S4 for crude monthly rate)

#### Monthly ESA use

**Fig. 1:**
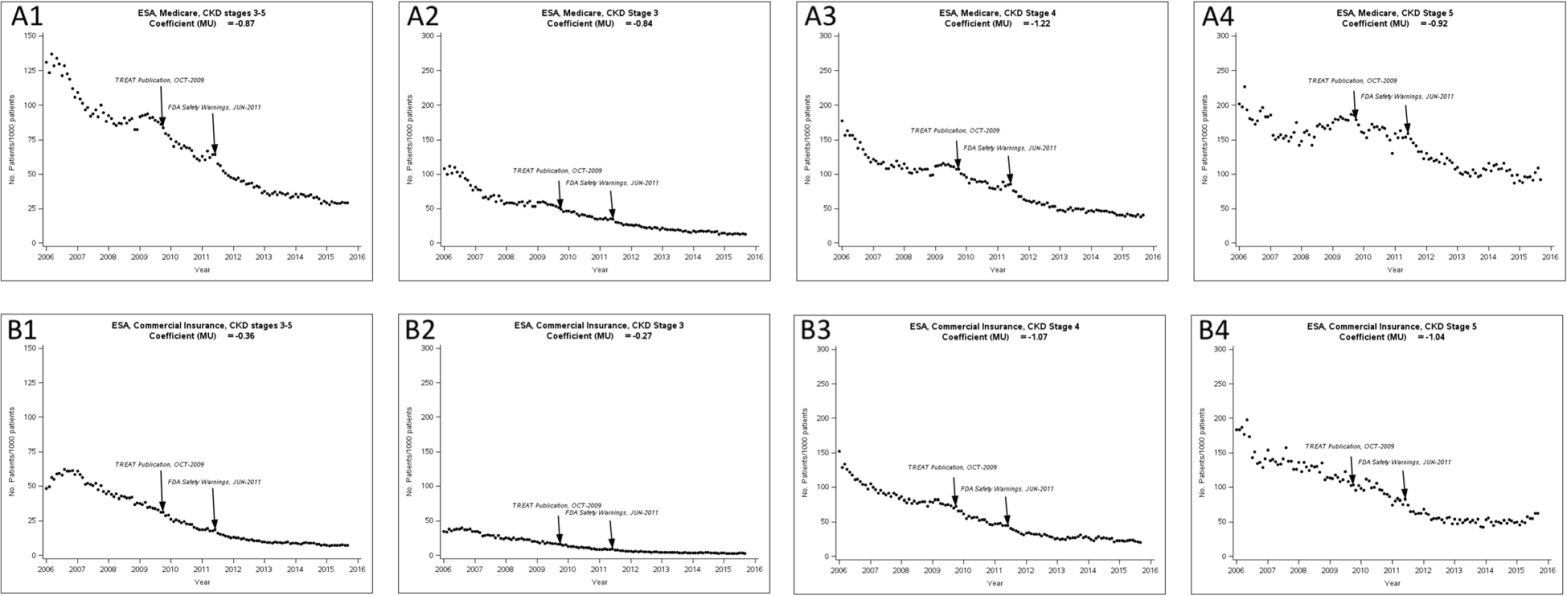
The use of erythropoiesis-stimulating agent (ESA) therapy in patients with chronic kidney disease (CKD) by insurance type and CKD stages. Coefficient MU: the mean difference in the monthly ESA. A1. Medicare CKD stages 3–5; A2. Medicare CKD stage 3; A3. Medicare CKD stage 4; A4. Medicare CKD stage 5. B1. Commercially insured CKD stages 3–5; B2. Commercially insured CKD stage 3; B3. Commercially insured CKD stage 4; B4. Commercially insured CKD stage5

Monthly ESA use are shown in Fig. 1 separately for CKD stages 3–5 in Medicare and commercially insured patients. Overall ESA use was 130.8 per 1000 Medicare patients in January 2006. After adjustment for seasonality, the mean difference (MU) in the monthly ESA use in Medicare patients was − 0.87‰ (*p* < 0.001), indicating on average, the ESA use decreased at a rate of 0.87 per 1000 patients per month over the study period, reaching 29.3 per 1000 patients in September 2015. Overall ESA prescribing prevalence was 48.3 per 1000 commercially insured patients in January 2006 and continuously decreased at a rate of 0.36 individuals per 1000 patients per month (MU = − 0.36%; *p* < 0.05), reaching 7.1 per 1000 patients in September 2015.

#### Monthly ESA use by CKD stages

We stratified our analysis for ESA use by CKD stages. The mean differences in monthly ESA use (MU) in Medicare patients showed that patients with CKD stage 4 had the largest decrease rate in the ESA use- CKD stages 3, 4 and 5 MU were − 0.84‰, − 1.21‰ and − 0.92‰, respectively. The mean differences in monthly ESA use in commercially insured patients showed patients with CKD stages 3 and 4 experienced a smaller decrease in the rate of ESA use than Medicare patients- CKD stages 3, 4, and 5 MU were − 0.27‰, − 1.07‰ and − 1.04‰, respectively.

#### Monthly intravenous Iron use

**Fig. 2:**
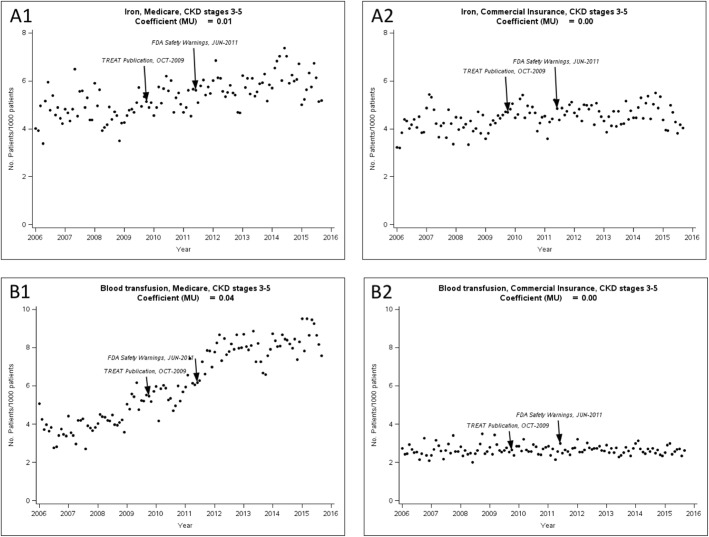
The use of (A) intravenous iron and (B) blood transfusions in patients with chronic kidney disease (CKD) by insurance type. Coefficient MU: the mean difference in the monthly iron and blood transfusions. A1. Medicare CKD stages 3–5; A2. Commercially insured CKD stages 3–5. B1. Medicare CKD stages 3–5; B2. Commercially insured CKD stages 3–5

Monthly intravenous iron supplementation and blood transfusion use are shown in Fig. 2 The mean of the monthly intravenous iron difference (MU) in Medicare patients was 0.01‰ (*p* = 0.435), indicating on average, the intravenous iron use increased, but this was not statistically significant and the rate was low (0.01 individuals per 1000 patients per month). Similarly, the mean of the monthly intravenous iron difference in commercially insured patients was 0.005‰ (*p* = 0.720).

#### Monthly blood transfusion use

The mean of the monthly blood transfusion use difference (MU) in Medicare patients was 0.03‰ (*p* = 0.120), indicating on average, the prevalence of blood transfusion in a latter month was not significantly different from a former month. The mean of the monthly blood transfusion difference in commercially insured patients was − 0.002‰ but this difference was not statistically significant (*p* = 0.957).

### Interrupted time series analysis using GEE model

Table 2 displays the probability of prescribing ESA, intravenous iron, and blood transfusions per month during the post-TREAT and post-FDA safety warning periods compared to the pre-TREAT period. After adjustment, the probabilities of prescribing ESA in the post-TREAT and post-FDA warning periods were 31% (odds ratio (OR) = 0.69, 95% confidence interval (CI): 0.67, 0.72) and 59% (OR = 0.41, 95% CI: 0.40, 0.42) lower than the pre-TREAT period for Medicare patients. After adjusting for covariates, the probability of prescribing intravenous iron was increased in the post-FDA warning period (OR = 1.11, 95% CI: 1.03–1.19). The probabilities of prescribing blood transfusion were increased by 14% (OR = 1.14, 95% CI: 1.06–1.23) and 31% (OR = 1.31, 95% CI: 1.22–1.39), respectively, during the post-TREAT and post-FDA warning periods compared to the pre-warning period.

**Table 2:** Probability of prescribing erythropoiesis-stimulating agent (ESA) and intravenous iron therapy, and blood transfusions during post-FDA safety warnings (June 2011–September 2015) vs pre-FDA warnings (January 2009–May 2011)

	ESA			Intravenous iron			Blood transfusions		
	Adjusted odds ratio	95% CI		Adjusted odds ratio	95% CI		Adjusted odds ratio	95% CI	
Medicare									
After TREAT published	0.69	0.67	0.72	1.03	0.94	1.12	1.14	1.06	1.23
After FDA warning	0.41	0.40	0.42	1.11	1.03	1.19	1.31	1.22	1.39
Female (vs male)	1.27	1.24	1.30	1.12	1.06	1.18	0.97	0.93	1.02
Age	1.01	1.01	1.01	0.99	0.99	0.99	1.01	1.01	1.02
CKD stage 4 (vs stage 3)	1.50	1.46	1.54	1.17	1.11	1.24	1.10	1.06	1.15
CKD stage 5 (vs stage 3)	2.22	2.13	2.31	1.39	1.25	1.54	1.41	1.30	1.53
Diabetes mellitus	1.22	1.19	1.27	0.93	0.87	1.00	0.79	0.75	0.83
Hypertension	0.90	0.88	0.92	1.01	0.94	1.10	1.00	0.95	1.06
Heart failure	0.90	0.88	0.93	1.12	1.05	1.19	1.35	1.29	1.42
Cerebrovascular disease	0.90	0.87	0.92	0.91	0.86	0.98	0.94	0.89	0.99
Peripheral artery disease	1.00	0.97	1.03	1.10	1.03	1.18	1.10	1.05	1.16
Chronic obstructive pulmonary disease	0.90	0.88	0.93	1.10	1.03	1.17	1.22	1.17	1.28
Charlson Comorbidity Index	1.00	1.00	1.01	1.09	1.07	1.11	1.17	1.15	1.18
Nephrologist involvement	1.33	1.31	1.37	1.00	0.95	1.06	0.97	0.93	1.01
Commercial insurance									
After TREAT published	0.55	0.53	0.57	1.04	0.98	1.10	0.93	0.87	1.00
After FDA warning	0.26	0.25	0.27	1.06	1.00	1.11	0.94	0.89	1.00
Female (vs male)	2.21	2.14	2.29	1.57	1.51	1.63	1.49	1.42	1.56
Age	1.00	1.00	1.01	0.99	0.99	0.99	0.99	0.99	1.00
CKD stage 4 (vs stage 3)	2.75	2.67	2.84	1.24	1.18	1.29	1.81	1.71	1.90
CKD stage 5 (vs stage 3)	4.34	4.15	4.56	1.40	1.29	1.54	2.12	1.92	2.34
Diabetes mellitus	1.39	1.34	1.45	0.96	0.91	1.00	0.71	0.67	0.76
Hypertension	1.01	0.98	1.04	0.98	0.94	1.03	1.06	1.00	1.13
Heart failure	1.05	1.00	1.10	1.10	1.03	1.18	1.79	1.68	1.91
Cerebrovascular disease	0.84	0.80	0.89	0.89	0.83	0.96	1.00	0.93	1.08
Peripheral artery Disease	0.97	0.92	1.03	1.16	1.08	1.24	1.27	1.18	1.37
Chronic obstructive pulmonary disease	0.87	0.84	0.91	1.31	1.24	1.39	1.10	1.04	1.18
Charlson Comorbidity Index	1.12	1.11	1.14	1.18	1.17	1.20	1.37	1.36	1.39
Nephrologist involvement	1.43	1.39	1.47	1.08	1.04	1.12	1.01	0.97	1.06

For Medicare patients, characteristics associated with the increased likelihood of ESA prescribing included advanced CKD stages, female, diabetes mellitus, and involvement of a nephrologist. Variables associated with the administration of intravenous iron included advanced CKD stages, female, heart failure, and higher CCI score. Older age, advanced CKD stages, heart failure, peripheral artery disease, COPD, and higher CCI scores were associated with an increased probability of receiving blood transfusions.

Similar results were observed in the commercially insured CKD patients. The probabilities of prescribing ESAs were 45 and 74% lower (OR = 0.55, 95% CI: 0.53–0.57 and OR = 0.26, 95% CI: 0.25–0.27) in the post-TREAT and post-FDA warning periods. The probabilities of prescribing intravenous iron were increased (OR = 1.04, 95% CI: 0.98–1.10 and OR = 1.06, 95% CI: 1.00–1.11). The probabilities of blood transfusions were 7% (OR = 0.93, 95% CI: 0.87–1.00) and 6% (OR = 0.94, 95% CI: 0.89–1.00) lower, respectively, during the post-TREAT and post-FDA warning periods compared to the pre-TREAT period. However, these changes were not statistically significant. Characteristics associated with the increased likelihood of prescribing ESA in this group included advanced CKD stages, being female, having diabetes mellitus, having a higher CCI score, and involvement of a nephrologist. Factors associated with an increased likelihood of prescribing intravenous iron included: being female, advanced CKD stages, having peripheral artery disease, COPD, a higher CCI score, and involvement of a nephrologist. Variables associated with associated with an increased probability of prescribing blood transfusions included: being female, advanced CKD stages, having heart failure, peripheral artery disease, COPD, and a higher CCI score.

## Discussion

The purposes of this study were to examine overall anemia management trends (ESA, intravenous iron and blood transfusion) in non-dialysis patients with CKD from 2006 to 2015, and to evaluate the impact of TREAT’s study results (October 2009) and FDA’s (June 2011) safety warnings and guidelines on the use of ESA therapy in the current treatment of anemia. We found that the use of ESA treatment in CKD non-dialysis patients decreased considerably from 2006 until 2015 (from 13 to 3% in Medicare patients; from 5 to 0.7% in commercially insured patients) in addition to finding a small increase in the use of intravenous iron supplementation (from 0.40 to 0.52% in Medicare patients; from 0.32 to 0.40% in commercially insured patients). We also found that the use of blood transfusions increased in Medicare patients (from 0.51 to 0.76% in Medicare patients) but not in commercially insured patients.

Interestingly, results of our time-series analysis indicated a steady decline in the use of EPO agents such that less than one fifth of patients received ESA in 2015 compared to 2005, with the trend starting as early as 2006, and the most significant changes occurring from 2006 to 2009. Notably, the initial decline in the use of ESA occurred with publication of the first randomized trials, CHOIR [5] and CREATE [6] in which investigators reported a higher prevalence of cardiovascular and cancer events in patients who received ESA agents [5, 6]. In addition, following closely after the publication of these two trial results, the KDOQI anemia guidelines were revised in 2007 to include an evidence-based warning to avoid hemoglobin levels above 13 g/dl when CKD patients are treated with ESAs [11]. The release of the 2007 recommendations corresponded with the largest drop in the use of ESA’s seen in this study and substantiate a previous study which also observed significant declines in the percentage of patients receiving ESAs between 2005 and 2009 [12].

Although we found slightly increase in the use of intravenous iron supplementation over the 10 years of the study, the proportion of patients who received intravenous iron remained small, at roughly 0.4–0.5% in 2015. In addition, we found that there was increase in the use of blood transfusions but the proportion of patients who received at least one transfusion remained relatively low, at 0.3–0.8% in 2015. Though there still remains questions as to when to initiate blood transfusions in patients with non-dialysis CKD, there is agreement that blood transfusions should be avoided in potential transplant candidates and used judiciously in all other patients [11]. As such, this would explain the very low rate of transfusions in this group of patients and only those that were older and sicker with advanced CKD were administered blood products. This finding is significant because there are concerns about an increase in blood transfusion as consequence of avoidance of ESAs. Transfusion avoidance is especially important in patients with advanced CKD because receipt of blood transfusions significantly increases the risk of developing allo-sensitization which may prolong the time on the kidney transplant waiting list and possibly jeopardize the kidney transplant outcome if one were to be transplanted [13, 14].

Similar to a previous study [15], our study found that after adjusting for clinical variables and co-morbidities using regression analyses, the proportion of CKD patients receiving at least one ESA decreased significantly after the publication of TREAT in 2009. After controlling for covariates, Medicare patients were significantly more likely to receive blood transfusions whereas there were no significant changes in commercially insured patients. We also explored anemia treatment patterns after the FDA warnings in 2011. To the best of our knowledge, no study has provided the impact of FDA actions yet. Similar to the trend after the TREAT publication, we found that the proportion of CKD patients treated with ESA’s continued to decrease significantly while there was an increase in the use of intravenous iron supplementations after the FDA warnings. Blood transfusion continued to increase significantly in Medicare patients while it remained stable in commercially insured patients after controlling for covariates.

These results suggest that in Medicare patients, the use of iron and blood transfusions substituted the use of ESAs to treat anemia associated with CKD. Interestingly, in our study, although changes in the use of ESAs were substantial, the use of iron and blood transfusions did not increase significantly in commercially-insured patients. Although the reason for this difference is unclear, it could reflect differences in clinical practice for elderly CKD patients with cardiovascular comorbidities as well as differences in insurance coverage for intravenous versus oral medications in addition to lower prevalence of anemia treatment in younger commercially-insured patients compared to older Medicare patients. It is worth noting that in 2012, the Center for Medicare and Medicaid Services (CMS) implemented changes to the prospective payment system, including ESA administration, became part of a capitated payment, incentivizing providers to administer minimum yet adequate amounts of expensive treatments while striving to provide quality care and improve patient outcomes [16]. Although this reimbursement change may have impacted on Medicare dialysis patients, it may influence providers’ practice for non-dialysis dependent CKD patients. A recent study reported that ESA use remained stable between 2006 and 2010, and then substantial declines in ESA use and hemoglobin levels occurred among patients on hemodialysis in the U.S. from 2010 to 2013, which reflects efforts in response to changes in FDA warning and reimbursement policy [17].

A striking finding was that among both groups of patients, the likelihood of receiving an ESA agent was greater if nephrologist was involved in the case (33–43% more likely to be prescribed ESA therapy). In contrast, a previous study found that that involvement of a nephrologist was associated with 18% lower likelihood of being prescribed ESA therapy during 2007–2011 [15]. Possible explanations for these discrepant findings could be differences in study populations and study periods. The previous study included cancer patients who receive ESA therapy due to cancer-related anemia [15]. Studies have shown that oncologists and hematologists were more likely than any other physician specialist including nephrologists to prescribe ESA [18, 19]. Our study, however, excluded patients with any cancer diagnosis to focus on CKD patients and their anemia management and was conducted for 2006–2015. However, the receipt of blood transfusions was not associated with being seen by a nephrologist. This may be due to better appreciation of the importance of transfusion avoidance by nephrologists.

A major strength of our study is the rigorous statistical analysis we used to determine the trends of anemia management overtime. The ARIMA model for estimating the monthly utilization patterns is popular because of the Box-Jenkins methodology in the modeling process which controls for secular trend (e.g, autocorrelation and seasonality) [20]. Specifically, the many events that occurred over the 10 year timespan of this study [CKD patients’ clinco-demographics, publication of a landmark clinical study (TREAT), and changes in FDA’s safety warnings for use of ESA’s], we used the interrupted time-series analysis GEE regression model that accounts for repeated measurement for the same patient adjusting for pertinent covariates- a valuable study design for evaluating longitudinal effects of population level interventions that have been implemented at a defined point in time [21–23].

There are also several study limitations. The first is the lack of hemoglobin data to determine whether the changes in practice led to changes in hemoglobin levels, to what extent, and if specific treatments were related to the severity of anemia. The revised EPO label provides more conservative dosing recommendations; however, we were unable to evaluate changes in EPO dose as information about EPO dose was no available in data. Oral iron was not included as it has variable coverage by insurance plans and is often purchased over the counter. In addition, this study included patients who had either commercial insurance or commercial plus Medicare supplemental insurance as their primary coverage. Thus, these results may not be generalizable to patients who are covered only by Medicare. This study is also limited by using ICD-9 codes, HCPCS, CPT, and revenue codes as recorded on administrative claims. It is possible that incomplete, missing, or miscoded claims impacted the study findings; however, coding errors are likely equally distributed across study periods and groups. Because we used the highest CKD stage during the 1-year baseline period for those who changed CKD stages over the study period, it is also possible that we might underestimate the lower stage and overestimate the higher stage. We also lacked patient clinical outcomes to determine how these prescribing patterns translated into patient outcomes. However, since there remains a lack of clarity and consensus within the current guidelines on how each of these therapeutic interventions should be used to treat anemia in CKD patients, interpretation of any findings would be difficult. But, it is clear from the data presented that physician prescribing patterns to correct anemia in CKD patients have changed with a noted decrease in the use of ESA’s and an increase in the use of intravenous iron supplementation and blood transfusions. These are important changes to debate because as healthcare providers, we need to weigh the possible benefits of using ESAs to avoid the need for blood transfusions against the increased risks for serious cardiovascular events. However, more work is clearly needed to better understand how these changes translate into patient and budgetary outcomes as well as to establish clear guidelines to help manage anemia in CKD patients.

## Conclusions

Over 10 years of study, there has been a marked decline in the number of CKD patients receiving ESA therapy, and consequently, a modest increase in the number of individuals receiving intravenous iron supplement and blood transfusions in patients with non-dialysis-dependent CKD. After the FDA warnings in 2011, ESA use continued to decrease and the iron supplement continued to increase. Blood transfusion continued to increase in Medicare patients but seemed to plateau in commercially-insured patients. Further studies are needed to evaluate the impact of these significant changes in anemia management on patient outcomes to include mortality, cardiovascular events, patient reported anemia symptoms and quality of life.

## Additional file

Additional file 1: **Table S1**. Codes used to identify claims of ESA, intravenous iron and blood transfusions. **Table S2**. ARIMA models for ESA, intravenous iron and blood transfusions. **Table S3**. Monthly rate of ESA, intravenous iron and blood transfusion in patients with CKD, Medicare Supplemental Database (per 1000 patients). **Table S4**. Monthly rate of ESA, intravenous iron and blood transfusion in patients with CKD, Commercially Insured Database (per 1000 patients). (DOCX 86 kb)

## Abbreviations

AIC: Akaike Infomration Criteria
AR: Autoregressive
ARIMA: Autoregressive Integrated Moving Average
CCI: Charlson comorbidity Index
CDC: Centers for Disease Prevention and Control
CHOIR: Correction of Hemoglobin and Outcomes in Renal Insufficiency
CKD: Chronic kidney disease
CMS: Center for Medicare and Medicaid Services
COPD: Chronic obstructive pulmonary disease
CPT: Clinical Procedural Terminology
CREATE: Cardiovascular Risk Reduction by Early Anemia Treatment with Epoetin Beta
ESAs: Erythropoiesis stimulating agents
FDA: Food and Drug Administration
GEE: Generalized Estimating Equation
HCPCS: Healthcare Common Procedure Coding System
KDOQI: National Kidney Disease Outcomes Quality Initiative
MA: Moving average
NDC: National Drug Code
TREAT: Trial to Reduced Cardiovascular Events with Ananesp Therapy
