# Supplementary material for: Trends in anemia care in non-dialysis-dependent chronic kidney disease (CKD) patients in the United States (2006–2015)
Source: BMC Nephrol. 2018 Nov 9;19:318. doi: 10.1186/s12882-018-1119-7 (PMC6230235; doi:10.1186/s12882-018-1119-7)
Supplement: Supplementary file 1 — Table S1. Codes used to identify claims of ESA, intravenous iron and blood transfusions. Table S2. ARIMA models for ESA, intravenous iron and blood transfusions. Table S3. Monthly rate of ESA, intravenous iron and blood transfusion in patients with CKD, Medicare Supplemental Database (per 1000 patients). Table S4. Monthly rate of ESA, intravenous iron and blood transfusion in patients with CKD, Commercially Insured Database (per 1000 patients). (DOCX 86 kb) [file 12882_2018_1119_MOESM1_ESM.docx]

**Table S1: Codes used to identify claims of ESA, intravenous iron and blood transfusion**

| Outcomes | Relevant codes | Measure description |
| --- | --- | --- |
| Use of ESA | HCPCS codes: Q0136, J0885, Q4055, Q4081, J0886, Q0137, J0880, J0881, Q4054, or J0882  NDC codes: 420230260xx, 548682523xx, 548685673xx, 555130126xx, 555130144xx, 555130148xx, 555130267xx, 555130283xx, 555130478xx, 555130823xx, 596760302xx, 596760303xx, 596760304xx, 596760310xx, 596760312xx, 596760320xx, 596760340xx, 635520478xx, 635520823xx, 54569313700, 54868580200, 246460090xx, 246460091xx, 246460092xx, 246460093xx, 246460094xx, 246460095xx, 246460096xx, 246460097xx, 548685429xx, 548685867xx, 555130002xx, 555130003xx, 555130004xx, 555130005xx, 555130006xx, 555130008xx, 555130010xx, 555130011xx, 555130012xx, 555130013xx, 555130013xx, 555130015xx, 555130021xx, 555130023xx, 555130025xx, 555130027xx, 555130028xx, 555130032xx, 555130039xx, 555130041xx, 555130043xx, 555130044xx, 555130046xx, 555130048xx, 555130053xx, 555130054xx, 555130057xx,555130090xx, 555130091xx, 555130092xx, 555130093xx, 555130094xx, 555130095xx, 555130096xx, 555130097xx, 555130110xx, 555130111xx, 597030043xx, 55513005804, 55513005801, 55513003704, 55513003701, 54868542800 | At least one ESA claim in patients with CKD  in the measurement month |
| Use of intravenous iron | HCPCS codes: J1750, J1760, J1770, J1780, J1751, J1752, J2915, J2916, S0098, W0231, J1755, Q0138, Q0139, or J3490 | At least one intravenous iron claim in patients with CKD in the measurement month |
| Blood transfusion | ICD-9 codes: 99.03-99.04  HCPCS/CPT codes: P9010, P9011, P9016, P9021, P9022, P9038, P9039, P9040, P9051, P9054, P9056, P9057, P9058, 36430  Revenue codes: 0380, 0381, 0382, 0391 | At least one blood transfusion claim in patients with CKD in the measurement month |

Table S2: ARIMA models for ESA, intravenous iron and blood transfusion use

|  | **p** | **d** | **q** | **s** | **MU** | **AR1,1** | **MA1,1** | **AR2,1** | **Constant** | **AIC** | **SBC** |
| --- | --- | --- | --- | --- | --- | --- | --- | --- | --- | --- | --- |
| **ESA** | | | | | | | | | | | |
| Medicare CKD stages 3-5 | **1** | **1** | **0** | **1** | **-0.87**** | **-0.33**** | **N/A** | **0.22** | **-0.90** | **604** | **196** |
| Medicare, CKD 3 | 1 | 1 | 0 | 1 | -0.84** | -0.41 | N/A | -0.30*** | -0.83 | 585 | 824 |
| Medicare, CKD 4 | 1 | 1 | 0 | 1 | -1.21** | -0.29** | N/A | 0.23* | -1.21 | 678 | 794 |
| Medicare, CKD 5 | 1 | 1 | 0 | 1 | -0.92 | -0.24* | N/A | 0.032 | -1.10 | 855 | 890 |
| Commercial insurance CKD stages 3-5 | 1 | 1 | 0 | 0 | **-0.36*** | **-0.32**** | **N/A** | **N/A** | **-0.47** | **437** | **443** |
| Commercial insurance, CKD 3 | 1 | 1 | 0 | 0 | -0.27** | -0.45*** | N/A | N/A | -0.40 | 361 | 366 |
| Commercial insurance, CKD 4 | 1 | 1 | 0 | 0 | -1.07* | 0.47** | N/A | N/A | -0.16 | 616 | 622 |
| Commercial insurance, CKD 5 | 1 | 1 | 0 | 0 | -1.04 | -0.32** | N/A | N/A | -1.38 | 810 | 816 |
| **Intravenous iron supplementation** | | | | | | | | | | | |
| Medicare stages CKD 3-5 | **0** | **1** | **1** | **0** | **0.01** | **N/A** | **0.74***** | **N/A** | **0.012** | **216** | **221** |
| Commercial insurance, CKD stages 3-5 | **0** | **1** | **1** | **0** | **0.005** | **N/A** | **0.69***** | **N/A** | **0.005** | **149** | **154** |
| **Blood transfusion** | | | | | | | | | | | |
| Medicare CKD stages 3-5 | 0 | 1 | 1 | 0 | 0.03 | N/A | 0.62*** | N/A | 0.035 | 223 | 228 |
| Commercial insurance CKD stages 3-5 | 1 | 1 | 0 | 1 | -0.002 | -0.32*** | N/A | 0.41*** | -0.001 | 63 | 72 |

Note: ARIMA model consists of three components (p, d, q) – order of autoregressive part (p), order of differencing (d), and order of moving average process (q): S: seasonality, MU: the mean term, AR1,1: parameter estimate for the first order of the autoregressive part; MA1,1: parameter estimate for the first order of the moving-average part; AR2,1: parameter estimate for the seasonality part; AIC: Akaike information criterion; SBC: Schwarz’s Bayesian criterion; *p<0.05 **p<0.01 ***p<0.001

**Table S3. Monthly rate of ESA, intravenous iron and blood transfusion in patients with CKD, Medicare Supplemental Database (per 1000 patients).**

|  |  | EPO | | | Intravenous Iron | | | Blood transfusion | | |
| --- | --- | --- | --- | --- | --- | --- | --- | --- | --- | --- |
| month | No. Pt | p | LL | UL | p | LL | UL | p | LL | UL |
| Jan-06 | 5733 | 130.8 | 122.1 | 139.6 | 4.0 | 2.4 | 5.6 | 5.1 | 3.2 | 6.9 |
| Feb-06 | 6106 | 123.3 | 115.1 | 131.6 | 3.9 | 2.4 | 5.5 | 4.3 | 2.6 | 5.9 |
| Mar-06 | 6460 | 136.8 | 128.5 | 145.2 | 5.0 | 3.2 | 6.7 | 3.7 | 2.2 | 5.2 |
| Apr-06 | 6809 | 128.7 | 120.7 | 136.6 | 3.4 | 2.0 | 4.8 | 4.0 | 2.5 | 5.5 |
| May-06 | 7155 | 134.0 | 126.1 | 141.9 | 5.2 | 3.5 | 6.8 | 3.6 | 2.2 | 5.0 |
| Jun-06 | 7565 | 129.7 | 122.1 | 137.2 | 5.9 | 4.2 | 7.7 | 3.8 | 2.4 | 5.2 |
| Jul-06 | 7943 | 121.1 | 113.9 | 128.3 | 4.8 | 3.3 | 6.3 | 2.8 | 1.6 | 3.9 |
| Aug-06 | 8168 | 128.4 | 121.2 | 135.7 | 5.4 | 3.8 | 7.0 | 2.8 | 1.7 | 4.0 |
| Sep-06 | 8509 | 122.5 | 115.5 | 129.4 | 4.6 | 3.1 | 6.0 | 3.4 | 2.2 | 4.6 |
| Oct-06 | 8789 | 118.7 | 111.9 | 125.4 | 4.9 | 3.4 | 6.4 | 3.8 | 2.5 | 5.0 |
| Nov-06 | 9219 | 111.8 | 105.4 | 118.3 | 4.4 | 3.1 | 5.8 | 3.5 | 2.3 | 4.7 |
| Dec-06 | 9482 | 105.6 | 99.4 | 111.8 | 4.2 | 2.9 | 5.5 | 3.4 | 2.2 | 4.5 |
| Jan-07 | 10391 | 109.1 | 103.1 | 115.1 | 4.8 | 3.5 | 6.1 | 4.4 | 3.2 | 5.7 |
| Feb-07 | 10700 | 104.4 | 98.6 | 110.2 | 4.7 | 3.4 | 6.0 | 3.6 | 2.4 | 4.7 |
| Mar-07 | 10856 | 101.6 | 95.9 | 107.3 | 4.3 | 3.1 | 5.6 | 3.4 | 2.3 | 4.5 |
| Apr-07 | 11206 | 96.9 | 91.4 | 102.4 | 4.8 | 3.5 | 6.1 | 2.9 | 1.9 | 3.9 |
| May-07 | 11398 | 98.4 | 93.0 | 103.9 | 6.5 | 5.0 | 8.0 | 4.2 | 3.0 | 5.4 |
| Jun-07 | 11707 | 92.2 | 86.9 | 97.4 | 4.5 | 3.3 | 5.7 | 4.2 | 3.0 | 5.4 |
| Jul-07 | 11872 | 93.8 | 88.5 | 99.0 | 5.6 | 4.2 | 6.9 | 4.3 | 3.1 | 5.5 |
| Aug-07 | 12166 | 96.7 | 91.4 | 101.9 | 5.6 | 4.3 | 6.9 | 2.7 | 1.8 | 3.6 |
| Sep-07 | 12487 | 91.7 | 86.6 | 96.8 | 4.9 | 3.7 | 6.1 | 3.9 | 2.8 | 5.0 |
| Oct-07 | 12655 | 100.2 | 95.0 | 105.4 | 5.3 | 4.0 | 6.6 | 3.8 | 2.7 | 4.9 |
| Nov-07 | 12806 | 94.7 | 89.6 | 99.8 | 4.4 | 3.2 | 5.5 | 3.7 | 2.6 | 4.7 |
| Dec-07 | 13046 | 88.2 | 83.4 | 93.1 | 4.4 | 3.2 | 5.5 | 3.8 | 2.8 | 4.9 |
| Jan-08 | 13364 | 92.6 | 87.6 | 97.5 | 5.9 | 4.6 | 7.2 | 4.0 | 3.0 | 5.1 |
| Feb-08 | 13505 | 90.3 | 85.4 | 95.1 | 5.0 | 3.8 | 6.1 | 4.5 | 3.4 | 5.6 |
| Mar-08 | 13646 | 86.6 | 81.9 | 91.3 | 5.6 | 4.4 | 6.9 | 4.4 | 3.3 | 5.5 |
| Apr-08 | 13764 | 85.4 | 80.7 | 90.0 | 3.9 | 2.9 | 5.0 | 4.4 | 3.3 | 5.5 |
| May-08 | 14033 | 86.9 | 82.3 | 91.6 | 4.1 | 3.0 | 5.1 | 4.2 | 3.1 | 5.3 |
| Jun-08 | 14149 | 86.7 | 82.1 | 91.4 | 4.2 | 3.1 | 5.2 | 4.2 | 3.1 | 5.2 |
| Jul-08 | 14280 | 90.7 | 86.0 | 95.4 | 4.9 | 3.8 | 6.0 | 4.5 | 3.4 | 5.6 |
| Aug-08 | 14364 | 87.2 | 82.5 | 91.8 | 4.4 | 3.3 | 5.5 | 4.0 | 2.9 | 5.0 |
| Sep-08 | 14471 | 89.0 | 84.4 | 93.6 | 4.7 | 3.6 | 5.8 | 3.9 | 2.9 | 5.0 |
| Oct-08 | 14684 | 90.3 | 85.7 | 94.9 | 4.6 | 3.5 | 5.7 | 4.1 | 3.1 | 5.1 |
| Nov-08 | 14875 | 82.4 | 78.0 | 86.8 | 3.5 | 2.5 | 4.4 | 4.2 | 3.2 | 5.3 |
| Dec-08 | 14858 | 82.3 | 77.9 | 86.7 | 4.2 | 3.2 | 5.3 | 3.6 | 2.6 | 4.5 |
| Jan-09 | 21173 | 91.7 | 87.8 | 95.6 | 4.3 | 3.4 | 5.1 | 5.1 | 4.1 | 6.0 |
| Feb-09 | 21771 | 92.3 | 88.5 | 96.2 | 4.5 | 3.7 | 5.4 | 4.8 | 3.9 | 5.7 |
| Mar-09 | 22417 | 93.0 | 89.2 | 96.8 | 4.8 | 3.9 | 5.7 | 5.6 | 4.6 | 6.6 |
| Apr-09 | 23040 | 93.7 | 89.9 | 97.4 | 4.8 | 3.9 | 5.7 | 5.4 | 4.5 | 6.4 |
| May-09 | 23629 | 90.8 | 87.1 | 94.4 | 4.7 | 3.8 | 5.6 | 6.2 | 5.2 | 7.2 |
| Jun-09 | 24100 | 91.1 | 87.4 | 94.7 | 5.1 | 4.2 | 6.0 | 4.8 | 3.9 | 5.6 |
| Jul-09 | 24637 | 89.0 | 85.5 | 92.6 | 5.7 | 4.8 | 6.7 | 5.2 | 4.3 | 6.1 |
| Aug-09 | 25099 | 87.9 | 84.4 | 91.4 | 4.9 | 4.1 | 5.8 | 5.2 | 4.3 | 6.1 |
| Sep-09 | 25510 | 86.1 | 82.6 | 89.5 | 5.3 | 4.4 | 6.2 | 5.5 | 4.6 | 6.4 |
| Oct-09 | 26104 | 83.9 | 80.5 | 87.2 | 5.1 | 4.3 | 6.0 | 5.5 | 4.6 | 6.4 |
| Nov-09 | 26600 | 79.3 | 76.1 | 82.6 | 4.9 | 4.0 | 5.7 | 5.2 | 4.3 | 6.1 |
| Dec-09 | 26905 | 78.3 | 75.1 | 81.6 | 5.1 | 4.2 | 5.9 | 5.7 | 4.8 | 6.6 |
| Jan-10 | 27688 | 75.5 | 72.4 | 78.6 | 4.6 | 3.8 | 5.3 | 6.0 | 5.1 | 6.9 |
| Feb-10 | 28246 | 70.2 | 67.3 | 73.2 | 4.9 | 4.1 | 5.7 | 4.2 | 3.4 | 4.9 |
| Mar-10 | 28383 | 73.6 | 70.5 | 76.6 | 5.7 | 4.9 | 6.6 | 5.8 | 5.0 | 6.7 |
| Apr-10 | 28642 | 71.8 | 68.8 | 74.8 | 5.1 | 4.2 | 5.9 | 6.0 | 5.1 | 6.9 |
| May-10 | 28863 | 68.8 | 65.9 | 71.8 | 5.7 | 4.8 | 6.5 | 5.9 | 5.0 | 6.8 |
| Jun-10 | 29163 | 70.4 | 67.4 | 73.3 | 6.2 | 5.3 | 7.1 | 5.3 | 4.4 | 6.1 |
| Jul-10 | 29312 | 69.3 | 66.4 | 72.2 | 5.6 | 4.7 | 6.4 | 5.4 | 4.5 | 6.2 |
| Aug-10 | 29594 | 68.7 | 65.8 | 71.6 | 6.0 | 5.1 | 6.9 | 4.7 | 3.9 | 5.5 |
| Sep-10 | 29827 | 67.3 | 64.4 | 70.1 | 4.7 | 3.9 | 5.5 | 5.0 | 4.2 | 5.8 |
| Oct-10 | 30014 | 63.0 | 60.2 | 65.7 | 5.3 | 4.5 | 6.1 | 6.0 | 5.1 | 6.9 |
| Nov-10 | 30144 | 61.5 | 58.8 | 64.2 | 5.5 | 4.7 | 6.3 | 5.2 | 4.4 | 6.0 |
| Dec-10 | 30217 | 59.9 | 57.2 | 62.5 | 5.0 | 4.2 | 5.8 | 5.7 | 4.8 | 6.5 |
| Jan-11 | 34505 | 62.9 | 60.3 | 65.4 | 4.7 | 4.0 | 5.4 | 5.9 | 5.1 | 6.8 |
| Feb-11 | 34844 | 60.6 | 58.1 | 63.1 | 4.9 | 4.1 | 5.6 | 6.6 | 5.7 | 7.4 |
| Mar-11 | 34936 | 66.8 | 64.2 | 69.4 | 5.6 | 4.8 | 6.4 | 7.4 | 6.5 | 8.3 |
| Apr-11 | 35121 | 62.3 | 59.7 | 64.8 | 4.5 | 3.8 | 5.2 | 6.2 | 5.3 | 7.0 |
| May-11 | 35299 | 64.1 | 61.6 | 66.7 | 5.7 | 4.9 | 6.4 | 6.1 | 5.3 | 6.9 |
| Jun-11 | 35593 | 64.0 | 61.5 | 66.6 | 5.6 | 4.8 | 6.4 | 6.2 | 5.4 | 7.0 |
| Jul-11 | 35929 | 57.6 | 55.2 | 60.1 | 5.1 | 4.4 | 5.8 | 6.3 | 5.5 | 7.1 |
| Aug-11 | 36121 | 56.4 | 54.0 | 58.7 | 5.8 | 5.0 | 6.6 | 7.3 | 6.4 | 8.1 |
| Sep-11 | 36330 | 52.6 | 50.3 | 54.9 | 6.0 | 5.2 | 6.8 | 6.6 | 5.8 | 7.4 |
| Oct-11 | 36267 | 50.6 | 48.3 | 52.9 | 5.4 | 4.6 | 6.2 | 7.9 | 6.9 | 8.8 |
| Nov-11 | 36325 | 49.2 | 46.9 | 51.4 | 5.8 | 5.0 | 6.5 | 7.8 | 6.9 | 8.7 |
| Dec-11 | 36290 | 47.8 | 45.6 | 50.0 | 5.5 | 4.7 | 6.2 | 7.0 | 6.1 | 7.9 |
| Jan-12 | 39322 | 47.0 | 45.0 | 49.1 | 6.0 | 5.3 | 6.8 | 7.8 | 6.9 | 8.7 |
| Feb-12 | 39764 | 45.9 | 43.9 | 48.0 | 6.8 | 6.0 | 7.7 | 8.2 | 7.4 | 9.1 |
| Mar-12 | 40292 | 47.2 | 45.1 | 49.2 | 6.1 | 5.4 | 6.9 | 8.7 | 7.8 | 9.6 |
| Apr-12 | 40298 | 45.0 | 42.9 | 47.0 | 6.1 | 5.3 | 6.9 | 7.3 | 6.5 | 8.2 |
| May-12 | 40641 | 45.1 | 43.1 | 47.1 | 5.6 | 4.8 | 6.3 | 8.5 | 7.6 | 9.4 |
| Jun-12 | 40957 | 42.8 | 40.9 | 44.8 | 5.3 | 4.6 | 6.1 | 7.6 | 6.8 | 8.5 |
| Jul-12 | 41215 | 43.0 | 41.1 | 45.0 | 5.5 | 4.8 | 6.2 | 7.8 | 7.0 | 8.7 |
| Aug-12 | 41329 | 43.7 | 41.7 | 45.7 | 5.8 | 5.1 | 6.5 | 8.2 | 7.3 | 9.1 |
| Sep-12 | 41748 | 40.5 | 38.6 | 42.4 | 5.5 | 4.8 | 6.2 | 7.9 | 7.1 | 8.8 |
| Oct-12 | 41991 | 41.3 | 39.4 | 43.2 | 5.4 | 4.7 | 6.1 | 8.7 | 7.8 | 9.6 |
| Nov-12 | 42258 | 40.7 | 38.8 | 42.6 | 4.7 | 4.0 | 5.3 | 8.0 | 7.1 | 8.8 |
| Dec-12 | 42717 | 36.5 | 34.7 | 38.3 | 4.7 | 4.0 | 5.3 | 8.0 | 7.2 | 8.9 |
| Jan-13 | 37922 | 37.8 | 35.9 | 39.7 | 6.2 | 5.4 | 7.0 | 8.7 | 7.8 | 9.6 |
| Feb-13 | 38294 | 36.1 | 34.2 | 37.9 | 5.7 | 5.0 | 6.5 | 8.1 | 7.2 | 9.0 |
| Mar-13 | 38675 | 34.6 | 32.8 | 36.5 | 6.1 | 5.3 | 6.9 | 7.9 | 7.0 | 8.8 |
| Apr-13 | 38650 | 36.0 | 34.2 | 37.9 | 5.5 | 4.7 | 6.2 | 8.1 | 7.2 | 9.0 |
| May-13 | 38832 | 37.4 | 35.5 | 39.3 | 6.1 | 5.3 | 6.9 | 8.9 | 8.0 | 9.8 |
| Jun-13 | 39087 | 35.0 | 33.2 | 36.8 | 5.4 | 4.6 | 6.1 | 7.3 | 6.4 | 8.1 |
| Jul-13 | 39009 | 36.8 | 34.9 | 38.7 | 5.5 | 4.8 | 6.3 | 8.2 | 7.3 | 9.1 |
| Aug-13 | 39310 | 36.1 | 34.3 | 38.0 | 5.9 | 5.1 | 6.6 | 7.3 | 6.4 | 8.1 |
| Sep-13 | 39347 | 35.0 | 33.2 | 36.9 | 5.9 | 5.2 | 6.7 | 6.7 | 5.9 | 7.5 |
| Oct-13 | 39400 | 36.0 | 34.1 | 37.8 | 6.3 | 5.5 | 7.1 | 6.6 | 5.8 | 7.4 |
| Nov-13 | 39208 | 32.9 | 31.1 | 34.7 | 5.2 | 4.4 | 5.9 | 7.6 | 6.7 | 8.4 |
| Dec-13 | 39028 | 33.7 | 32.0 | 35.5 | 5.8 | 5.1 | 6.6 | 7.9 | 7.0 | 8.8 |
| Jan-14 | 34853 | 35.5 | 33.5 | 37.4 | 5.7 | 4.9 | 6.5 | 8.7 | 7.7 | 9.7 |
| Feb-14 | 34887 | 33.2 | 31.3 | 35.1 | 6.5 | 5.7 | 7.4 | 8.4 | 7.4 | 9.3 |
| Mar-14 | 34829 | 35.3 | 33.3 | 37.2 | 6.8 | 6.0 | 7.7 | 8.1 | 7.1 | 9.0 |
| Apr-14 | 34860 | 35.2 | 33.3 | 37.1 | 7.0 | 6.2 | 7.9 | 8.1 | 7.1 | 9.0 |
| May-14 | 33736 | 33.9 | 32.0 | 35.9 | 6.0 | 5.2 | 6.8 | 8.7 | 7.7 | 9.7 |
| Jun-14 | 33815 | 34.1 | 32.2 | 36.0 | 7.4 | 6.5 | 8.3 | 8.5 | 7.5 | 9.4 |
| Jul-14 | 33849 | 35.2 | 33.3 | 37.2 | 7.0 | 6.1 | 7.9 | 8.4 | 7.4 | 9.4 |
| Aug-14 | 33932 | 33.2 | 31.2 | 35.1 | 5.9 | 5.1 | 6.7 | 8.2 | 7.2 | 9.2 |
| Sep-14 | 34001 | 33.2 | 31.3 | 35.1 | 6.2 | 5.4 | 7.1 | 8.0 | 7.0 | 8.9 |
| Oct-14 | 34049 | 32.1 | 30.2 | 34.0 | 6.0 | 5.2 | 6.8 | 8.5 | 7.5 | 9.4 |
| Nov-14 | 34141 | 28.8 | 27.0 | 30.6 | 6.1 | 5.2 | 6.9 | 7.4 | 6.5 | 8.3 |
| Dec-14 | 34518 | 30.4 | 28.6 | 32.3 | 6.7 | 5.9 | 7.6 | 8.3 | 7.4 | 9.3 |
| Jan-15 | 24999 | 29.4 | 27.3 | 31.5 | 5.0 | 4.1 | 5.9 | 9.5 | 8.3 | 10.7 |
| Feb-15 | 25064 | 28.0 | 26.0 | 30.1 | 5.2 | 4.3 | 6.1 | 7.8 | 6.7 | 8.9 |
| Mar-15 | 25007 | 29.9 | 27.8 | 32.0 | 5.6 | 4.7 | 6.6 | 9.5 | 8.3 | 10.7 |
| Apr-15 | 24964 | 29.0 | 27.0 | 31.1 | 6.3 | 5.3 | 7.3 | 8.7 | 7.5 | 9.8 |
| May-15 | 24918 | 28.9 | 26.8 | 30.9 | 5.7 | 4.8 | 6.7 | 9.5 | 8.3 | 10.7 |
| Jun-15 | 24787 | 28.8 | 26.7 | 30.8 | 6.7 | 5.7 | 7.8 | 9.3 | 8.1 | 10.5 |
| Jul-15 | 24431 | 29.7 | 27.5 | 31.8 | 6.1 | 5.2 | 7.1 | 8.6 | 7.5 | 9.8 |
| Aug-15 | 24110 | 29.4 | 27.2 | 31.5 | 5.1 | 4.2 | 6.0 | 8.2 | 7.0 | 9.3 |
| Sep-15 | 23891 | 29.3 | 27.2 | 31.4 | 5.2 | 4.3 | 6.1 | 7.6 | 6.5 | 8.7 |

**Table S4. Monthly rate of ESA, intravenous iron and blood transfusion in patients with CKD, Commercially Insured Database (per 1000 patients).**

|  |  | EPO | | | Intravenous Iron | | | Blood transfusion | | |
| --- | --- | --- | --- | --- | --- | --- | --- | --- | --- | --- |
| month | No. Pt | p | LL | UL | p | LL | UL | p | LL | UL |
| Jan-06 | 14265 | 48.3 | 44.8 | 51.8 | 3.2 | 2.3 | 4.2 | 2.7 | 1.9 | 3.6 |
| Feb-06 | 14087 | 49.5 | 46.0 | 53.1 | 3.2 | 2.3 | 4.1 | 2.4 | 1.6 | 3.2 |
| Mar-06 | 13801 | 56.4 | 52.5 | 60.2 | 3.8 | 2.8 | 4.9 | 2.5 | 1.6 | 3.3 |
| Apr-06 | 13680 | 55.1 | 51.3 | 58.9 | 4.4 | 3.3 | 5.5 | 2.9 | 2.0 | 3.8 |
| May-06 | 13412 | 58.8 | 54.8 | 62.8 | 4.3 | 3.2 | 5.4 | 2.7 | 1.8 | 3.6 |
| Jun-06 | 13230 | 59.3 | 55.2 | 63.3 | 4.0 | 2.9 | 5.1 | 2.5 | 1.6 | 3.3 |
| Jul-06 | 12980 | 58.0 | 54.0 | 62.0 | 4.2 | 3.1 | 5.3 | 2.5 | 1.7 | 3.4 |
| Aug-06 | 12547 | 61.9 | 57.7 | 66.1 | 4.4 | 3.2 | 5.5 | 2.2 | 1.3 | 3.0 |
| Sep-06 | 12295 | 60.7 | 56.5 | 64.9 | 4.1 | 2.9 | 5.2 | 2.4 | 1.6 | 3.3 |
| Oct-06 | 11973 | 61.1 | 56.8 | 65.3 | 4.5 | 3.3 | 5.7 | 3.3 | 2.2 | 4.3 |
| Nov-06 | 12289 | 61.1 | 56.9 | 65.3 | 3.8 | 2.7 | 4.9 | 2.4 | 1.5 | 3.2 |
| Dec-06 | 12441 | 58.3 | 54.2 | 62.4 | 3.9 | 2.8 | 4.9 | 2.1 | 1.3 | 2.9 |
| Jan-07 | 17695 | 60.9 | 57.3 | 64.4 | 4.9 | 3.8 | 5.9 | 2.4 | 1.7 | 3.1 |
| Feb-07 | 18259 | 58.3 | 54.9 | 61.7 | 5.4 | 4.4 | 6.5 | 2.7 | 1.9 | 3.4 |
| Mar-07 | 18626 | 56.4 | 53.1 | 59.7 | 5.3 | 4.3 | 6.4 | 3.2 | 2.4 | 4.0 |
| Apr-07 | 19190 | 51.0 | 47.9 | 54.1 | 4.8 | 3.8 | 5.8 | 2.9 | 2.1 | 3.6 |
| May-07 | 19655 | 51.9 | 48.8 | 55.0 | 4.2 | 3.3 | 5.1 | 2.6 | 1.9 | 3.3 |
| Jun-07 | 20242 | 51.3 | 48.2 | 54.3 | 3.7 | 2.8 | 4.5 | 2.2 | 1.5 | 2.8 |
| Jul-07 | 20662 | 50.2 | 47.3 | 53.2 | 4.1 | 3.2 | 5.0 | 2.6 | 1.9 | 3.3 |
| Aug-07 | 21200 | 52.1 | 49.1 | 55.1 | 4.2 | 3.4 | 5.1 | 3.0 | 2.2 | 3.7 |
| Sep-07 | 21769 | 47.1 | 44.3 | 49.9 | 3.6 | 2.8 | 4.4 | 2.5 | 1.8 | 3.1 |
| Oct-07 | 22033 | 50.2 | 47.3 | 53.0 | 4.8 | 3.9 | 5.7 | 3.4 | 2.6 | 4.2 |
| Nov-07 | 22559 | 46.0 | 43.3 | 48.7 | 4.2 | 3.4 | 5.1 | 2.6 | 1.9 | 3.2 |
| Dec-07 | 22940 | 44.2 | 41.5 | 46.9 | 3.4 | 2.6 | 4.1 | 2.6 | 1.9 | 3.2 |
| Jan-08 | 24104 | 46.1 | 43.4 | 48.7 | 4.5 | 3.6 | 5.3 | 2.8 | 2.2 | 3.5 |
| Feb-08 | 24515 | 43.8 | 41.2 | 46.3 | 4.0 | 3.2 | 4.7 | 2.3 | 1.7 | 2.9 |
| Mar-08 | 24864 | 42.1 | 39.7 | 44.6 | 4.5 | 3.6 | 5.3 | 2.6 | 2.0 | 3.2 |
| Apr-08 | 25148 | 44.5 | 41.9 | 47.0 | 4.1 | 3.3 | 4.8 | 2.4 | 1.8 | 3.0 |
| May-08 | 25722 | 41.1 | 38.7 | 43.5 | 4.2 | 3.4 | 5.0 | 2.5 | 1.9 | 3.1 |
| Jun-08 | 26015 | 42.5 | 40.1 | 45.0 | 3.3 | 2.6 | 4.0 | 2.0 | 1.5 | 2.5 |
| Jul-08 | 26089 | 42.0 | 39.6 | 44.5 | 4.3 | 3.5 | 5.1 | 2.5 | 1.9 | 3.1 |
| Aug-08 | 26392 | 41.3 | 38.9 | 43.7 | 3.9 | 3.2 | 4.7 | 2.6 | 2.0 | 3.2 |
| Sep-08 | 26700 | 41.3 | 38.9 | 43.7 | 4.0 | 3.2 | 4.8 | 3.0 | 2.3 | 3.6 |
| Oct-08 | 27141 | 41.9 | 39.5 | 44.2 | 4.7 | 3.9 | 5.5 | 3.5 | 2.8 | 4.2 |
| Nov-08 | 27627 | 36.6 | 34.3 | 38.8 | 3.8 | 3.1 | 4.5 | 2.5 | 1.9 | 3.0 |
| Dec-08 | 27726 | 38.1 | 35.8 | 40.3 | 4.6 | 3.8 | 5.4 | 2.6 | 2.0 | 3.2 |
| Jan-09 | 36046 | 37.6 | 35.6 | 39.5 | 3.6 | 3.0 | 4.2 | 2.8 | 2.3 | 3.3 |
| Feb-09 | 36749 | 36.7 | 34.8 | 38.6 | 3.8 | 3.2 | 4.4 | 2.4 | 1.9 | 2.9 |
| Mar-09 | 37748 | 38.4 | 36.5 | 40.4 | 4.2 | 3.5 | 4.8 | 3.4 | 2.9 | 4.0 |
| Apr-09 | 38894 | 34.8 | 33.0 | 36.7 | 4.3 | 3.7 | 5.0 | 2.9 | 2.4 | 3.5 |
| May-09 | 39955 | 34.9 | 33.1 | 36.7 | 4.2 | 3.6 | 4.9 | 2.6 | 2.1 | 3.1 |
| Jun-09 | 40793 | 34.1 | 32.3 | 35.8 | 4.6 | 3.9 | 5.2 | 2.5 | 2.1 | 3.0 |
| Jul-09 | 42182 | 34.0 | 32.3 | 35.7 | 4.4 | 3.8 | 5.1 | 2.6 | 2.1 | 3.1 |
| Aug-09 | 43255 | 33.1 | 31.4 | 34.7 | 4.6 | 3.9 | 5.2 | 2.8 | 2.3 | 3.3 |
| Sep-09 | 44252 | 31.4 | 29.7 | 33.0 | 4.7 | 4.1 | 5.3 | 2.5 | 2.1 | 3.0 |
| Oct-09 | 45225 | 31.4 | 29.8 | 33.0 | 4.7 | 4.1 | 5.3 | 2.7 | 2.2 | 3.1 |
| Nov-09 | 46142 | 28.6 | 27.1 | 30.1 | 4.8 | 4.2 | 5.5 | 2.4 | 1.9 | 2.8 |
| Dec-09 | 46959 | 29.1 | 27.6 | 30.6 | 5.0 | 4.4 | 5.7 | 2.8 | 2.4 | 3.3 |
| Jan-10 | 46649 | 26.1 | 24.6 | 27.5 | 4.5 | 3.9 | 5.1 | 2.9 | 2.4 | 3.3 |
| Feb-10 | 47277 | 24.5 | 23.1 | 25.8 | 4.6 | 4.0 | 5.2 | 2.6 | 2.1 | 3.1 |
| Mar-10 | 47743 | 25.7 | 24.3 | 27.2 | 5.3 | 4.6 | 5.9 | 3.2 | 2.7 | 3.7 |
| Apr-10 | 48474 | 24.8 | 23.5 | 26.2 | 5.4 | 4.8 | 6.1 | 2.7 | 2.2 | 3.1 |
| May-10 | 48966 | 23.6 | 22.2 | 24.9 | 4.5 | 3.9 | 5.1 | 2.6 | 2.1 | 3.0 |
| Jun-10 | 49395 | 24.3 | 23.0 | 25.7 | 5.0 | 4.3 | 5.6 | 2.6 | 2.1 | 3.0 |
| Jul-10 | 50099 | 22.9 | 21.6 | 24.2 | 4.7 | 4.1 | 5.3 | 2.9 | 2.5 | 3.4 |
| Aug-10 | 50433 | 22.5 | 21.2 | 23.8 | 4.9 | 4.3 | 5.5 | 2.8 | 2.4 | 3.3 |
| Sep-10 | 51083 | 22.3 | 21.1 | 23.6 | 4.7 | 4.1 | 5.2 | 2.4 | 2.0 | 2.9 |
| Oct-10 | 51380 | 20.4 | 19.1 | 21.6 | 3.9 | 3.4 | 4.4 | 2.4 | 2.0 | 2.8 |
| Nov-10 | 51822 | 19.6 | 18.4 | 20.8 | 4.2 | 3.7 | 4.8 | 2.7 | 2.3 | 3.1 |
| Dec-10 | 52201 | 18.4 | 17.3 | 19.6 | 4.5 | 3.9 | 5.1 | 2.8 | 2.3 | 3.2 |
| Jan-11 | 56127 | 18.5 | 17.4 | 19.6 | 4.5 | 4.0 | 5.1 | 2.8 | 2.4 | 3.3 |
| Feb-11 | 56089 | 18.7 | 17.6 | 19.9 | 3.6 | 3.1 | 4.1 | 2.4 | 2.0 | 2.8 |
| Mar-11 | 56416 | 19.3 | 18.2 | 20.5 | 4.3 | 3.8 | 4.8 | 2.7 | 2.3 | 3.1 |
| Apr-11 | 56809 | 18.0 | 16.9 | 19.0 | 4.4 | 3.9 | 5.0 | 2.1 | 1.8 | 2.5 |
| May-11 | 57051 | 17.8 | 16.7 | 18.9 | 5.2 | 4.6 | 5.8 | 2.6 | 2.1 | 3.0 |
| Jun-11 | 57507 | 18.5 | 17.4 | 19.6 | 4.8 | 4.3 | 5.4 | 3.0 | 2.5 | 3.4 |
| Jul-11 | 58006 | 16.2 | 15.2 | 17.2 | 4.4 | 3.8 | 4.9 | 2.5 | 2.1 | 2.9 |
| Aug-11 | 58223 | 15.3 | 14.3 | 16.3 | 4.9 | 4.3 | 5.4 | 2.6 | 2.2 | 3.1 |
| Sep-11 | 58704 | 14.9 | 13.9 | 15.9 | 4.6 | 4.0 | 5.1 | 2.6 | 2.2 | 3.0 |
| Oct-11 | 59035 | 13.9 | 12.9 | 14.8 | 4.7 | 4.2 | 5.3 | 2.4 | 2.0 | 2.8 |
| Nov-11 | 59125 | 13.3 | 12.4 | 14.3 | 5.0 | 4.4 | 5.6 | 2.7 | 2.3 | 3.2 |
| Dec-11 | 59194 | 12.7 | 11.8 | 13.6 | 5.1 | 4.5 | 5.7 | 2.8 | 2.3 | 3.2 |
| Jan-12 | 67382 | 13.1 | 12.3 | 14.0 | 4.7 | 4.2 | 5.2 | 3.2 | 2.8 | 3.6 |
| Feb-12 | 67408 | 12.8 | 11.9 | 13.6 | 4.5 | 4.0 | 5.0 | 2.5 | 2.2 | 2.9 |
| Mar-12 | 67906 | 12.6 | 11.7 | 13.4 | 4.8 | 4.3 | 5.3 | 2.5 | 2.2 | 2.9 |
| Apr-12 | 68006 | 11.9 | 11.1 | 12.7 | 4.3 | 3.8 | 4.8 | 2.7 | 2.3 | 3.0 |
| May-12 | 68231 | 12.3 | 11.5 | 13.1 | 5.0 | 4.5 | 5.5 | 3.0 | 2.6 | 3.4 |
| Jun-12 | 68569 | 11.6 | 10.8 | 12.4 | 5.0 | 4.5 | 5.5 | 2.8 | 2.4 | 3.2 |
| Jul-12 | 68689 | 11.1 | 10.3 | 11.9 | 4.8 | 4.3 | 5.4 | 2.7 | 2.3 | 3.1 |
| Aug-12 | 68967 | 11.6 | 10.8 | 12.4 | 5.0 | 4.4 | 5.5 | 2.7 | 2.4 | 3.1 |
| Sep-12 | 69314 | 10.4 | 9.6 | 11.1 | 4.2 | 3.7 | 4.6 | 2.7 | 2.3 | 3.1 |
| Oct-12 | 69416 | 10.8 | 10.0 | 11.5 | 5.1 | 4.5 | 5.6 | 2.8 | 2.4 | 3.2 |
| Nov-12 | 69970 | 10.5 | 9.7 | 11.2 | 4.7 | 4.2 | 5.2 | 2.6 | 2.3 | 3.0 |
| Dec-12 | 70251 | 9.5 | 8.8 | 10.2 | 4.5 | 4.0 | 5.0 | 2.6 | 2.2 | 3.0 |
| Jan-13 | 52491 | 9.6 | 8.8 | 10.5 | 4.3 | 3.8 | 4.9 | 2.9 | 2.5 | 3.4 |
| Feb-13 | 52989 | 9.2 | 8.4 | 10.0 | 3.8 | 3.3 | 4.4 | 2.5 | 2.1 | 2.9 |
| Mar-13 | 53372 | 9.3 | 8.5 | 10.1 | 4.5 | 3.9 | 5.1 | 2.7 | 2.3 | 3.2 |
| Apr-13 | 53582 | 8.8 | 8.0 | 9.6 | 4.1 | 3.6 | 4.7 | 2.5 | 2.1 | 2.9 |
| May-13 | 54202 | 9.6 | 8.8 | 10.4 | 4.7 | 4.1 | 5.3 | 2.8 | 2.3 | 3.2 |
| Jun-13 | 54428 | 9.4 | 8.6 | 10.2 | 4.1 | 3.6 | 4.6 | 2.3 | 1.9 | 2.7 |
| Jul-13 | 54728 | 9.4 | 8.5 | 10.2 | 4.7 | 4.2 | 5.3 | 2.4 | 2.0 | 2.8 |
| Aug-13 | 55218 | 9.0 | 8.2 | 9.8 | 4.2 | 3.6 | 4.7 | 2.5 | 2.1 | 2.9 |
| Sep-13 | 55442 | 9.2 | 8.4 | 10.0 | 4.2 | 3.7 | 4.8 | 2.8 | 2.3 | 3.2 |
| Oct-13 | 55675 | 9.9 | 9.0 | 10.7 | 5.2 | 4.6 | 5.7 | 2.6 | 2.2 | 3.0 |
| Nov-13 | 55879 | 8.5 | 7.8 | 9.3 | 4.4 | 3.9 | 5.0 | 2.3 | 1.9 | 2.7 |
| Dec-13 | 56148 | 8.7 | 8.0 | 9.5 | 4.8 | 4.2 | 5.3 | 2.7 | 2.3 | 3.2 |
| Jan-14 | 58256 | 8.8 | 8.0 | 9.6 | 4.5 | 3.9 | 5.0 | 3.0 | 2.5 | 3.4 |
| Feb-14 | 58305 | 8.5 | 7.7 | 9.2 | 4.5 | 3.9 | 5.0 | 3.1 | 2.7 | 3.6 |
| Mar-14 | 58200 | 8.2 | 7.5 | 8.9 | 4.9 | 4.3 | 5.4 | 2.7 | 2.3 | 3.1 |
| Apr-14 | 58234 | 8.7 | 7.9 | 9.4 | 5.3 | 4.7 | 5.9 | 2.5 | 2.1 | 3.0 |
| May-14 | 58426 | 9.1 | 8.4 | 9.9 | 4.5 | 3.9 | 5.0 | 2.5 | 2.1 | 2.9 |
| Jun-14 | 58697 | 8.8 | 8.1 | 9.6 | 5.1 | 4.5 | 5.6 | 2.7 | 2.3 | 3.1 |
| Jul-14 | 59255 | 8.8 | 8.1 | 9.6 | 5.4 | 4.8 | 6.0 | 2.6 | 2.2 | 3.0 |
| Aug-14 | 59553 | 8.7 | 8.0 | 9.5 | 4.4 | 3.9 | 4.9 | 2.7 | 2.3 | 3.1 |
| Sep-14 | 59890 | 8.2 | 7.5 | 8.9 | 5.0 | 4.5 | 5.6 | 2.5 | 2.1 | 2.9 |
| Oct-14 | 60303 | 8.2 | 7.5 | 9.0 | 5.5 | 4.9 | 6.1 | 2.7 | 2.2 | 3.1 |
| Nov-14 | 61125 | 7.1 | 6.4 | 7.8 | 4.9 | 4.3 | 5.4 | 2.4 | 2.0 | 2.8 |
| Dec-14 | 61939 | 7.6 | 6.9 | 8.2 | 5.3 | 4.8 | 5.9 | 2.3 | 1.9 | 2.7 |
| Jan-15 | 44986 | 7.4 | 6.6 | 8.2 | 4.4 | 3.8 | 5.0 | 2.5 | 2.0 | 3.0 |
| Feb-15 | 45276 | 6.9 | 6.1 | 7.7 | 4.0 | 3.4 | 4.5 | 2.9 | 2.4 | 3.4 |
| Mar-15 | 45643 | 7.0 | 6.3 | 7.8 | 3.9 | 3.3 | 4.5 | 3.0 | 2.5 | 3.5 |
| Apr-15 | 45980 | 7.2 | 6.4 | 7.9 | 5.0 | 4.3 | 5.6 | 2.4 | 2.0 | 2.9 |
| May-15 | 46385 | 7.3 | 6.5 | 8.1 | 4.7 | 4.1 | 5.3 | 2.6 | 2.1 | 3.0 |
| Jun-15 | 46660 | 7.4 | 6.6 | 8.2 | 4.3 | 3.7 | 4.9 | 2.7 | 2.2 | 3.1 |
| Jul-15 | 47058 | 7.6 | 6.8 | 8.3 | 3.8 | 3.2 | 4.4 | 2.7 | 2.2 | 3.2 |
| Aug-15 | 47251 | 7.3 | 6.6 | 8.1 | 4.2 | 3.6 | 4.8 | 2.3 | 1.9 | 2.8 |
| Sep-15 | 47498 | 7.1 | 6.4 | 7.9 | 4.0 | 3.5 | 4.6 | 2.6 | 2.2 | 3.1 |
